# Supplementary figures and images for: A literature review on the analysis of symptom-based clinical pathways: Time for a different approach?
Source: PLOS Digit Health. 2022 May 26;1(5):e0000042. doi: 10.1371/journal.pdig.0000042 (PMC9931260; doi:10.1371/journal.pdig.0000042)

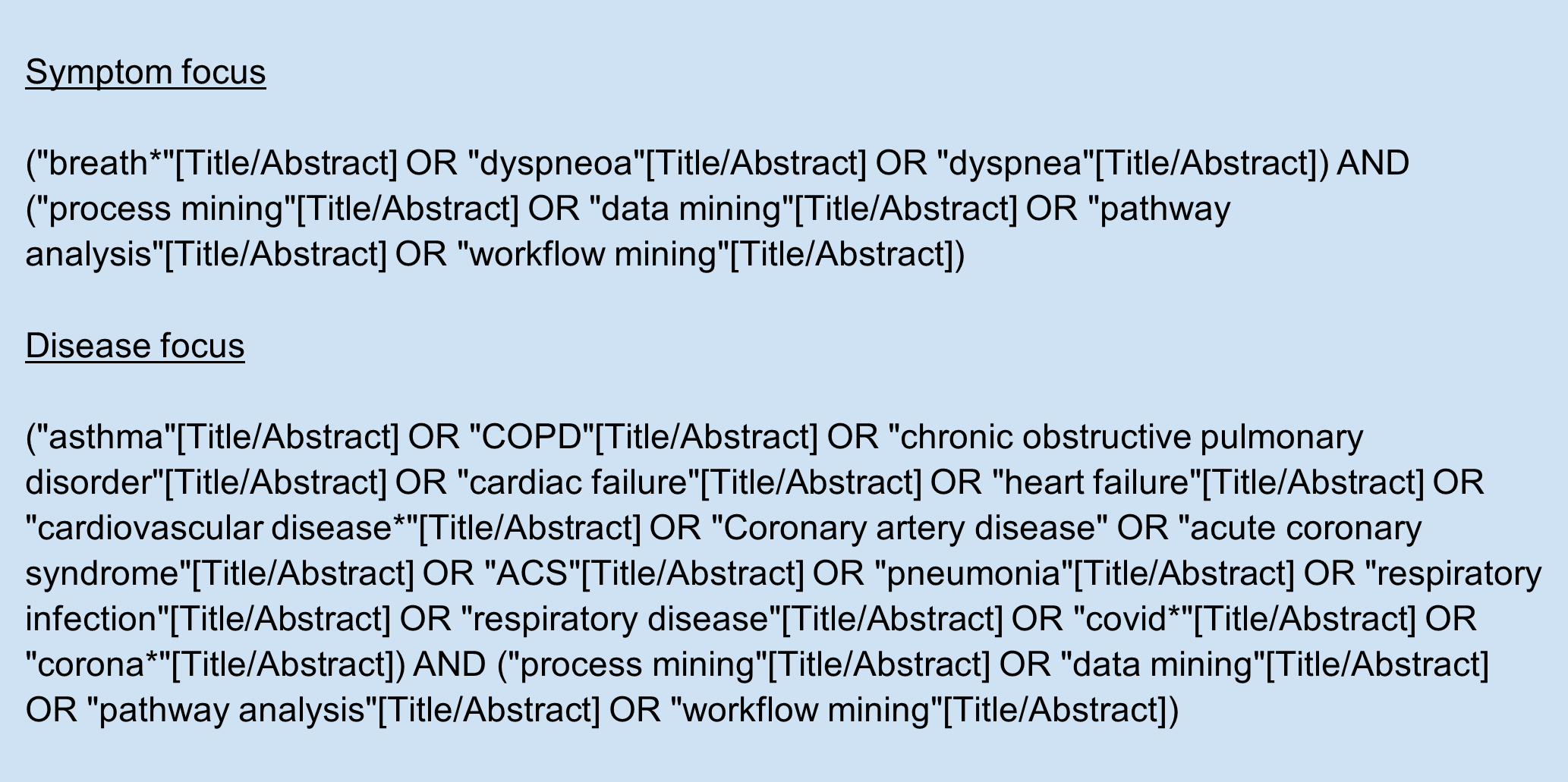

Supplement: S1 Appendix — (TIF) [file pdig.0000042.s001.tif]

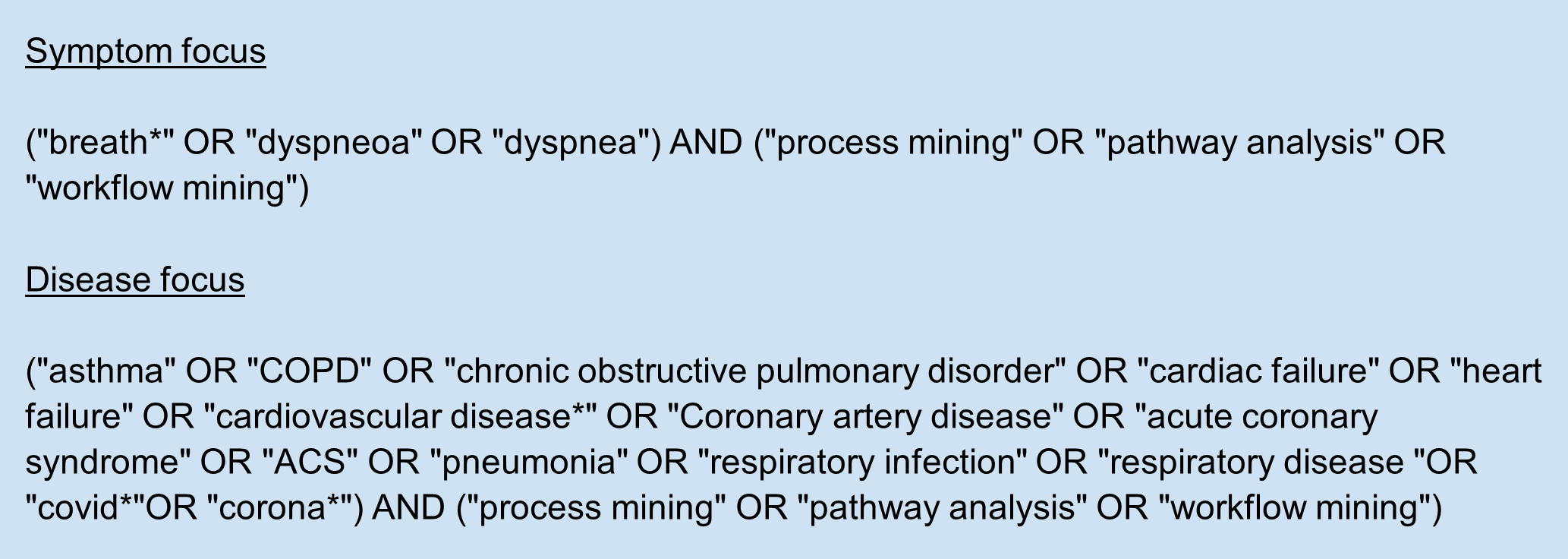

Supplement: S2 Appendix — To make the search work on IEEE Xplore and ACM Digital Library, the [Title/Abstract] restriction was removed. To counter a significant increase in search results, the search term ‘Data mining’ was also removed. (TIF) [file pdig.0000042.s002.tif]
