# Supplementary material for: A literature review on the analysis of symptom-based clinical pathways: Time for a different approach?
Source: PLOS Digit Health. 2022 May 26;1(5):e0000042. doi: 10.1371/journal.pdig.0000042 (PMC9931260; doi:10.1371/journal.pdig.0000042)
Supplement: Attachment — Submitted filename: Response to Reviewers.pdf [file pdig.0000042.s004.pdf]

Dr Nammunikankanange Janak Gunatilleke
The University of Edinburgh,
Edinburgh, UK.

22nd February 2022.

Dear Henry,

Revisions following review | A literature review on the analysis of symptom-based clinical pathways: Time for a different approach?

Thank you for reviewing our manuscript and for the opportunity to submit a revision, informed by the constructive review comments. We were pleased that the reviewers described our work as 'valuable' and 'clearly presented'. We have considered and incorporated many of the helpful comments and suggestions made.

A point-by-point summary of our responses to reviewer comments and our revisions are included below.

Review comment or suggestion	Author response	Location of revision
**Reviewer 1**		
The authors did a literature review to identify examples of process mining methodology applied to breathlessness clinical pathways. This can be considered a healthcare specialty. So the number of the included studies can be normal. They encountered two process mining studies in this area in the literature. Also, they synthesized retrospective analysis studies by applying data mining, NLP, AI techniques to the healthcare data. Thanks for this valuable review.<br><br>The authors did not follow any literature study guidelines. They have not already named this study as a systematic literature review. But they should define the research question for this review. It makes the hard to follow the results.<br><br>I have two major concerns: 1) the presentation ways of the results and 2) the related work section is missing.	We thank the reviewer for their helpful comments and are pleased that they found this to be a valuable review.<br><br>As noted by the reviewer, we have deliberately not used the term systematic literature review as we did not fully adopt this methodology, but we have focused on studies relevant for clinical pathways of patients with breathlessness.<br><br>We thank the reviewer for their suggestion to set out a clear research question and we have added this to the introduction section – "In this study we sought to answer the question – has process mining or related techniques been used to analyse clinical pathways for patients presenting with breathlessness?"	Lines 128 to 129
Authors should present the results in a more structured way. Authors can define research questions and report the answers to these questions or add new fields to the	We agree that further structure to the results table would add value and make it easier for the reader to follow the results.	

results table. The summary of findings column could not be easily followed even if the authors discussed the results in the discussion part.	In order to enable this, we have done the following:<br><br>• added the following column to the summary table – 'Techniques and methods used' to make this key information clearer to readers<br><br>• expanded the 'summary of key findings' to provide additional relevant detail. We do not wish to overwhelm the readers within a summary table, but believe there is now sufficient information presented to follow the logic of each included study.<br><br>• divided the summary table into two sections covering symptom and disease focussed studies.<br><br>• as suggested by the other reviewer, we have also elaborated on the rationale for the project at the end of the 'Introduction' section, which helps place the results in better context.	Results table - Table 1: Summary of included studies<br><br>'Introduction' section – lines 128 to 133
Also, in the summary and findings column, there are some techniques such as Hierarchical Clustering Analysis, random forest analyses, etc. Maybe you create a new field as techniques/methods or a new research question. Also, you must give references for each technique.	Thank you for this helpful suggestion. We have added the following column to the summary table – 'Techniques and methods used'.<br><br>Whilst we fully appreciate the reviewer's perspective on referencing individual techniques, we have chosen not to include these in the summary table. This is because the exact methodological approach used within each of the listed studies is not necessarily accurately represented by a generic reference on that technique. We feel that to include a reference within the summary table would risk misrepresenting the methods within these studies. However, where we have discussed methods in more detail in the discussion, we have included wider referencing of this e.g. Heuristic Miner and Miner-infrequent, and we have added the standard reference textbook for these techniques (reference 33, cited in line 235)	Results table - Table 1: Summary of included studies
Also, a new field as "contribution facet" can be added to this table. There are comparison papers, methodological papers, etc.	All of our studies included in the final list were primary research papers analysing datasets so we do not believe that a contribution facet would be meaningful in the summary table. However, we have taken on board this feedback and	Line 168

	adjusted the methods to make this clearer.	
You may group the findings of the papers according to the types of the used techniques or the contribution types.	Thank you for the helpful suggestion. The used techniques unfortunately do not provide a helpful means of grouping the studies. However, we have removed the ‘Symptom or Disease focus?’ column and have grouped the table separately by ‘Symptom’ and ‘Disease’ focussed papers.	Results table - Table 1: Summary of included studies
You may remove the title column for the primary studies.	We have removed the column from the summary table.	Results table - Table 1: Summary of included studies
You should report separately primary and secondary studies. You discussed the secondary studies in the discussion part. But it would be better that you place a new section as related work. You should mention the differences and core issues of these papers and refer to your contributions from these studies.	We do not fully understand the reviewer’s point regarding primary and secondary studies.<br><br>Additional studies in the discussion are included to place our work in context of the wider field of process mining and related techniques, but these studies would not meet the inclusion criteria set out in the methods and are therefore not reported within our results. We think it is important that the discussion integrates the findings of our literature review with related work, rather than simply report the existence of other studies. We have therefore chosen not to force a separation in the manner suggested.<br><br>We have made this clearer in the revised manuscript with the addition of a statement to clarify where discussed studies would not meet our inclusion criteria.	Line 350
Line 124: Give the references for algorithms: alpha miner, heuristic miner, and inductive miner.	We have added references for the relevant techniques and added fuzzy miner as a technique that is commonly used.	Line 121
Line 126: This paragraph can be short, maybe you can merge it with the previous one, or firstly you can mention the four dimensions of the quality of the modes as fitness, precision, generalization, and simplicity. After these sentences, you can give an explanation for each dimension with the questions sentences.	We have merged the paragraph with the previous one as suggested by the reviewer.	Lines 112 to 126
Line174: Maybe you can refer to snowballing techniques with complementary searching.	We have now included ‘forward citations’, one of the main techniques we used, as an example to elaborate. This point is also highlighted in the ‘Search strategy’ sub section.	Line 177

Missing the explanations for the acronym “NHS” (Line 88) and “COPD” (192)	We have added the explanations for the two acronyms where appropriate.	Lines – 85 (NHS) and within final results table (COPD)
Line 187. There was a grammar error as “. [17]”.	We have updated the sentence.	Line 191 (now reference 18)
Do not use the digits for the number which are smaller than 10	We have updated the relevant instances as suggested.	Whole manuscript
Use consistent usage for "et al."	We have updated the relevant instances.	Results table - Table 1: Summary of included studies
**Reviewer 2**		
Thank you for the opportunity to review this paper. It is a valuable topic that deserves discussion in the literature.<br>The manuscript is sound and clearly presented. Some points for consideration for revision:	We thank the reviewer for their helpful comments and are pleased that they found this to be a valuable topic and a clearly presented manuscript.	Not applicable
The review is looking to 'synthesise the evidence for the application of process mining to the analysis of clinical pathways for patients with breathlessness'. Since the review focused on additional techniques, with only one of the studies included using process mining, this could be revisited and clarified.	We agree with the need for clarification and in additional response to reviewer 1, we have set out a clear research question referencing process mining and related techniques within the revised ‘Introduction’ section.	Lines 128 to 129
At the end of the Introduction, please consider elaborating on why was this project conducted and what was the rationale for this study?	Thank you for this suggestion. We have added content to elaborate these points and highlight the importance of the topic and research area. This section also now includes a clearer research question.	‘Introduction’ section – lines 128 to 133
In the methods section, please include details of any methodological quality appraisals implemented in the review.	As we did not perform a formal systematic literature review, we did not undertake formal quality assessment using a structured tool. However, the reviewer raises an important point, and we have used opportunities to highlight quality of included studies throughout the manuscript. In the Results summary table, we have included assessments of sample size and generalisability in the ‘summary of key findings’.<br><br>We have also highlighted limitations of the studies within our discussion, but have added additional detail to suggest how future studies could overcome the methodological limitations of included studies.	Results table - Table 1: Summary of included studies<br><br>Lines 313 to 321

In the results section, the table was valuable in describing and identifying key aspects and results from each of the reviewed studies. However, an interpretation of the results of these studies and their value to the reader is lacking and it would be advantageous to provide the reader with this critical narrative and analysis for the studies included. As you have concluded that process mining 'has clear potential application to this area', I would suggest incorporating your interpretation and assessment of the final studies included in the review with a conclusion about their value, impact and potential for directing future research for clinical pathways for patients with breathlessness.	Thank you for your valuable feedback. The other reviewer also noted a lack of some study context within the summary table. We have made the following changes:<br>• separated techniques and methods used in a separate column of the table for ease of reading.<br>• expanded 'summary of key findings' - to focus on more key results from included papers. We have tried to find a balance between detail and overwhelming a reader looking at a summary table and literature review.<br>• as suggested, we have elaborated the 'Limitations and future research' section with suggestions for future research to develop a stronger evidence base from larger, multicentre cohort studies with external validation of models.	Results table - Table 1: Summary of included studies<br><br>Lines 321 to 324

We believe the changes made have strengthened the submission and hope the Reviewers and Editors agree.

Thank you for your continuing consideration of this manuscript.

Yours sincerely,

Dr Nammunikankanange Janak Gunatilleke | *Dr Atul Anand* | *Dr Jacques Fleuriot*
