# Supplementary material for: A literature review on the analysis of symptom-based clinical pathways: Time for a different approach?
Source: PLOS Digit Health. 2022 May 26;1(5):e0000042. doi: 10.1371/journal.pdig.0000042 (PMC9931260; doi:10.1371/journal.pdig.0000042)
Supplement: Attachment — Submitted filename: review.docx [file pdig.0000042.s003.docx]

The authors did a literature review to identify examples of process mining methodology applied to breathlessness clinical pathways. This can be considered a healthcare specialty. So the number of the included studies can be normal. They encountered two process mining studies in this area in the literature. Also, they synthesized retrospective analysis studies by applying data mining, NLP, AI techniques to the healthcare data. Thanks for this valuable review.

The authors did not follow any literature study guidelines. They have not already named this study as a systematic literature review. But they should define the research question for this review. It makes the hard to follow the results.

I have two major concerns: 1) the presentation ways of the results and 2) the related work section is missing.

 1) Authors should present the results in a more structured way. Authors can define research questions and report the answers to these questions or add new fields to the results table. The summary of findings column could not be easily followed even if the authors discussed the results in the discussion part.

Also, in the summary and findings column, there are some techniques such as HierarchicalClusteringAnalysis,	random forest analyses, etc. Maybe you create a new field as techniques/methods or a new research question. Also, you must give references for each technique.

Also, a new field as “contribution facet” can be added to this table. There are comparison papers, methodological papers, etc.

You may group the findings of the papers according to the types of the used techniques or the contribution types.

You may remove the title column for the primary studies.

2) You should report separately primary and secondary studies. You discussed the secondary studies in the discussion part. But it would be better that you place a new section as related work. You should mention the differences and core issues of these papers and refer to your contributions from these studies.

Also, there are some minor issues:

Line 124: Give the references for algorithms: alpha miner, heuristic miner, and inductive miner.

Line 126: This paragraph can be short, maybe you can merge it with the previous one, or firstly you can mention the four dimensions of the quality of the modes as fitness, precision, generalization, and simplicity. After these sentences, you can give an explanation for each dimension with the questions sentences.

Line174: Maybe you can refer to snowballing techniques with complementary searching.

Missing the explanations for the acronym “NHS” (Line 88) and “COPD” (192)

Line 187. There was a grammar error as “. [17]”.

Whole document:

Do not use the digits for the number which are smaller than 10

Use consistent usage for "et al."
