# Supplementary material for: A literature review on the analysis of symptom-based clinical pathways: Time for a different approach?
Source: PLOS Digit Health. 2022 May 26;1(5):e0000042. doi: 10.1371/journal.pdig.0000042 (PMC9931260; doi:10.1371/journal.pdig.0000042)
Supplement: Attachment — Submitted filename: Response to Reviewers.docx [file pdig.0000042.s005.docx]

Dr Nammunikankanange Janak Gunatilleke
The University of Edinburgh,
Edinburgh, UK.

25th March 2022.

Dear Henry,

**Revisions following review | A literature review on the analysis of symptom-based clinical pathways: Time for a different approach?**

Thank you for reviewing our revised manuscript and for the opportunity to submit a revision. We were pleased that the reviewers feel that the majority of their comments have been addressed and that reviewer 1 feels that the manuscript is ready for publishment. We have considered and incorporated the two suggestions made by reviewer 2.

A point-by-point summary of our responses to reviewer comments and our revisions are included below.

| **Review comment or suggestion** | **Author response** | **Location of revision** |
| --- | --- | --- |
| **Reviewer 2** | | |
| Research questions can be better worded for a more open-ended approach to the research e.g. utilizing 'how, why, what, which..' instead of 'has..' | We thank the reviewer for their helpful comment and have updated the research question accordingly. | Line 128 |
| As the manuscript did not include methodological quality appraisal, this can be mentioned in limitations of the study. | We agree with the suggestion and have included a relevant statement in the ‘Limitations and future research’ section. | Lines 320 to 322 |

We have also reviewed the references and have made the following two changes:

1. Line 215 – changed reference 15 to 16. We have also taken the opportunity to correct a minor typo regarding the number of studies related to this reference – from 69 to 67
2. Line 254 - changed reference 19 to 20

We believe the changes made have strengthened the submission and hope the Reviewers and Editors agree.

Thank you for your continuing consideration of this manuscript.

Yours sincerely,

*Dr Nammunikankanange Janak Gunatilleke | Dr Atul Anand | Dr Jacques Fleuriot*
